# Recent evaluation about inflammatory mechanisms in nonalcoholic fatty liver disease

**DOI:** 10.3389/fphar.2023.1081334

**Published:** 2023-03-16

**Authors:** Chong Song, Xian Long, Jianbin He, Yongpan Huang

**Affiliations:** ^1^ Medicine School, Changsha Social Work College, Changsha, Hunan, China; ^2^ Department of Respiratory and Critical Care Medicine, The First People’s Hospital of Huaihua, Affiliated to University of South China, Huaihua, Hunan, China

**Keywords:** non-alcoholic fatty liver diseases, non-alcoholic steatohepatitis, inflammation, peroxisome proliferator-activated receptor- γ (PPAR-γ), insulin resistance

## Abstract

Non-alcoholic fatty liver disease (NAFLD) is common chronic metabolic liver disorder which is associated with fat accumulation in the liver. It causes a wide range of pathological effects such as insulin resistance, obesity, hypertension, diabetes, non-alcoholic steatohepatitis (NASH) and cirrhosis, cardiovascular diseases. The molecular mechanisms that cause the initiation and progression of NAFLD remain fully unclear. Inflammation is regarded as a significant mechanism which could result in cell death and tissue injury. Accumulation of leukocytes and hepatic inflammation are important contributors in NAFLD. Excessive inflammatory response can deteriorate the tissue injury in NAFLD. Thus, inhibition of inflammation improves NAFLD by reducing intrahepatic fat content, increasing β-oxidation of fatty acids, inducing hepato-protective autophagy, overexpressing peroxisome proliferator-activated receptor- γ (PPAR-γ), as well as attenuating hepatocyte apoptosis and increasing insulin sensitivity. Therefore, understanding the molecules and signaling pathways suggests us valuable information about NAFLD progression. This review aimed to evaluate the inflammation in NAFLD and the molecular mechanism on NAFLD.

## 1 Introduction

Non-alcoholic fatty liver disease (NAFLD) is one of the most common chronic metabolic liver diseases recognized globally, with a prevalence of 25.2% (Ye et al., 2020). NAFLD includes a variety of benign reversible pathological changes including liver steatosis, and could also progress to more severe and irreversible pathological changes (Cobbina and Akhlaghi, 2017). 24% of patients can develop non-alcoholic steatohepatitis (NASH) and Cirrhosis; less than 15% of patients may eventually develop liver failure and hepatocellular carcinoma from cirrhosis (Schwabe et al., 2020). Non-alcoholic fatty liver disease (NAFLD) is a prominent cause of liver-related morbidity and mortality (Younossi et al., 2016; Albhaisi and Noureddin, 2021). Non-alcoholic steatohepatitis (NASH), the advanced form of NAFL, is illustrated by steatosis, inflammation, hepatocyte injury (e.g., ballooning), with or without fibrosis, which could progress to cirrhosis, fibrosis, liver-related complications and even hepatocellular carcinoma, which is seriously life-threatening (Huby and Gautier, 2022). Much progress has been made in understanding the pathogenesis of NASH and the complex and multifactorial (Hardy et al., 2016; Machado and Diehl, 2016) molecular pathways during development and progression. It has attracted more and more attention from scholars at home and abroad. It is currently believed that the mechanism of liver inflammation is an important driving force for the occurrence and development of NASH. This paper intended to provide an overview of the recent advances in this related field.

## 2 Extrahepatic factors in NASH

**FIGURE 1:**
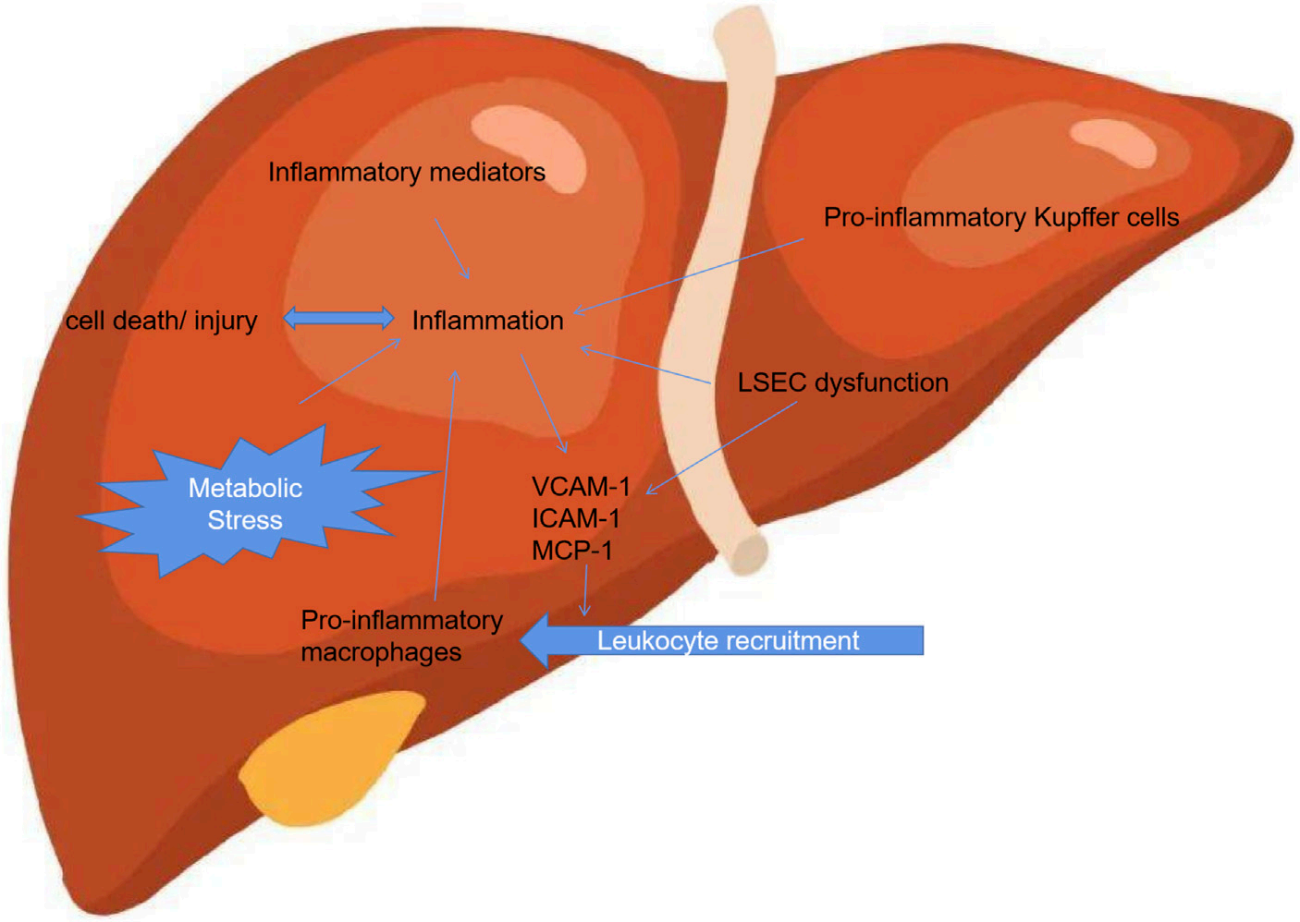
Inflammation associated with NAFLD.

NASH is considered the progressive form of NAFLD and is characterized by liver steatosis, inflammation, hepatocellular injury and different degrees of fibrosis (Schuster et al., 2018). Accumulating studies demonstrated that a pivotal issue relates to the identification of those factors that trigger inflammation, thus fuelling the transition from NAFLD to NASH (Guo et al., 2022; An et al., 2023). These triggers of inflammtion in liver might be associated with their origins outside the liver as well as inside the organ, both of which accelerate to NASH development (Han et al., 2022). Although the marked progress in unravelling the mechanism that underlie NAFLD development and progression has made, yet the elucidation of its mechanism about the pathogenesis remains limited. Thus, the identification of aberrent factors that trigger inflammation, thereby preceding the transition from NAFLD to NASH. Several lines of studies have demonstrated that hepatic inflammatory response is regarded as an important driving force during the hepatic progression. Furthermore, extrahepatic factors including adipose tissue dysfunction and the gut could trigger the inflammation and exacerbate the tissue injury (Figure 1).

### 2.1 Increased carbohydrate and fat intake

A large number of studies have demonstrated that fructose could act as a pro-inflammatory factor, up-regulating liver inflammatory genes, reducing liver mitochondrial β-oxidation and ATP (Adenosine Triphosphate) levels, causing metabolic dysfunction and promoting the development of NASH (Lim et al., 2010; Rom et al., 2020). Increased intake of high fructose is associated with liver fibrosis, dyslipidemia, endoplasmic reticulum stress, hepatocyte ballooning, inflammation, and impaired insulin signaling (Jensen et al., 2018; Pouwels et al., 2022).

Increased intake of dietary saturated fatty acids induces *de novo* fat synthesis, promoting endoplasmic reticulum stress and cell apoptosis. Saturated fatty acids are lipotoxic to the liver and could cause NASH in mice alone or in combination with a high-fructose diet (Arrese et al., 2016; Lastuvkova et al., 2021). Free cholesterol in liver samples from NASH patients was higher than healthy controls. The results showed that the liver in a cholesterol diet-induced NASH mouse model exhibited mitochondrial dysfunction, increased reactive oxygen species (ROS) due to excessive lipid accumulation, and endoplasmic reticulum stress, and the inclusion of hepatocytes (Al-Rasadi et al., 2015). A series of chain reactions such as activation of the death pathway induce a strong inflammatory response (Arrese and Karpen, 2010; Asrih and Jornayvaz, 2013). Furthermore, accumulation of free cholesterol in Kupffer cells and hepatic stellate cells activates inflammation and promotes fibrosis. Conversely, removing cholesterol from a high-fat and high-cholesterol diet in mice reduced plasma VLDL levels and prevented liver inflammation and ballooning (Al-Rasadi et al., 2015). Linoleic acid, the most abundant n-6 polyunsaturated fatty acid in the Western diet, is the immediate precursor to oxidized linoleic acid metabolites, a group of bioactive lipids (Van Name et al., 2020). Lowering dietary linoleic acid levels can reduce the synthesis and/or accumulation of oxidized linoleic acid metabolites in humans. Oxidative metabolites from arachidonic acid and linoleic acid are associated with markers of steatosis, liver injury, and insulin sensitivity, and the relevant oxidative metabolites are more abundant in the plasma of NASH patients than NAFLD patients (Civelek and Podszun, 2022; Gao et al., 2022). These data suggest that oxidized linoleic acid metabolites play a crucial role in NASH.

### 2.2 Insulin resistance and adipose tissue and inflammatory factors

NASH-related metabolic dysfunction is closely associated with insulin resistance and is a major driver of steatosis (Parthasarathy et al., 2020). Progressive adipose tissue dysfunction and insulin resistance may be key factors promoting the development of NASH. Visceral adipose tissue-released macrophages produced chemokines and cytokines involved in inflammation and fibrosis in human NASH. In a mouse model of NASH, mRNA levels of adipose tissue macrophage-related and inflammation-related genes were increased (Osorio-Conles et al., 2021). Adipose tissue dysfunction could lead to the accumulation of inflammatory cells in visceral adipose tissue, causing liver damage and the secretion of pro-inflammatory related mediators, including tumor necrosis factor (TNF), interleukin-1β (IL-1β) and IL-6; in turn, these inflammations promoted impaired insulin signaling (Daryabor et al., 2020). Adipose tissue insulin resistance was higher in NASH patients than in patients with simple steatosis. Furthermore, adipose tissue insulin resistance was associated with liver and muscle insulin resistance and liver fibrosis (Maximos et al., 2015; Rotman and Neuschwander-Tetri, 2017). The results also confirmed that obesity-associated inflammation induces insulin resistance (IR), which is central to NAFLD or hepatic steatosis. Prediction of HOMA values by SCGF-β levels, likely mediated by markers of inflammation, characterises a recent study, shedding some light on mechanisms inducing/worsening IR of male patients with obesity-related NAFLD (Tarantino et al., 2020).

As one of the largest endocrine organs in the body, adipose tissue could secrete inflammatory factors such as TNF and IL-6, as well as adipokines such as adiponectin and leptin, which further drove the progression of NASH. In the collected serum of NASH patients, it could be found that the content of leptin and adiponectin *in vivo* is negatively correlated (Zhao et al., 2018; Fan et al., 2020). In addition, adiponectin could inhibit hepatic steatosis, reduce insulin resistance, and has anti-inflammatory, anti-apoptotic and insulin-sensitizing functions. Furthermore, in addition to promoting inflammation, TNF may also increase insulin resistance and promote NASH progression. IL-6 promotes insulin resistance through multiple mechanisms, and NASH severity, fibrosis stage, and insulin resistance are associated. The study found that leptin in the early stage of NASH may prevent hepatic steatosis by inhibiting *de novo* synthesis of intrahepatic fat. However, as the disease progressed, leptin has pro-inflammatory and pro-fibrotic abilities (Jiménez-Cortegana et al., 2021). Therefore, leptin may have a bidirectional effects on NASH. However, this hypothesis has not been tested in humans.

### 2.3 The gut-liver axis

The occurrence of NASH is related to intestinal flora imbalance and intestinal barrier dysfunction. The body regulates intestinal barrier permeability through the expression of CX3C-chemokine receptor 1 (CX3CR1). Studies have shown that deletion of CX3CR1 impairs intestinal barrier function and exacerbates the occurrence of NASH in a mouse model (Schneider et al., 2019; Ni et al., 2022). In addition, junctional adhesion molecule A expression levels were decreased and mucosal inflammation was increased in the colonic mucosa of NASH patients. In mice lacking junctional adhesion molecule A, increased intestinal permeability enables bacterial translocation to the liver and drives NASH. Therefore, disruption of intestinal barrier integrity may predispose patients to hepatic inflammation and contribute to the development of NASH (Zhang et al., 2020). Whether intestinal permeability is responsible for endotoxin exposure is unclear, but endotoxin induces an inflammatory response by activating inflammatory cells in the liver. Studies have found that NASH patients have higher blood endotoxin levels than healthy individuals (Sinakos et al., 2022). However, whether the permeability of the gut epithelium to gut microbial endotoxins is diet-induced has not been established. Furthermore, topical intestinal anti-inflammatory drugs fed mice on a high-fat diet reduced intestinal inflammation while improving intestinal permeability and hepatic steatosis (She et al., 2022). However, studies addressing the association between endotoxemia and the histological features of NASH remain controversial (Nagata et al., 2022; Wang et al., 2022). Therefore, more accurate biomarkers are needed to rigorously assess the contribution of endotoxemia to NASH. Gut microbes could be influenced by diet, which varies from person to person and over time. Therefore, more research is needed to reveal the association between the gut microbiome and NASH.

Bile acids are involved in the development of NAFLD and NASH (Jia et al., 2021). First, bile acids have important regulatory effects on lipid and carbohydrate metabolism, and if dysregulated, it could lead to glucose and lipid imbalances in the body and promote inflammation and fibrosis (Sheng et al., 2022). In addition, bile acids and gut microbiota were interrelated. On the one hand, changes in bile acid metabolism could affect the composition of the microbiota; on the other hand, gut dysbiosis affects bile acid composition and promoted the development of NAFLD and NASH. Defects in hepatic bile acid transport have been found in experimental models of NASH, and cholestasis may occur, leading to liver injuries and inflammation (van Golen et al., 2018). Several studies have found that NASH patients had higher levels of bile acids than NAFLD, and that altered bile acid metabolism was associated with the histological features of NASH (Abdelmalek, 2021). Therefore, alterations in bile acid metabolism may play a pro-inflammatory role in the pathogenesis of NASH, but the mechanism are needed to be investigated in further studies.

## 3 Intrahepatic factors in NASH

### 3.1 Oxidative stress in hepatocytes

Oxidative stress is a common feature of chronic liver disease and plays a critical role in NASH progression. Oxidative stress markers correlate with neutrophil number and degree of liver injury. ROS disrupts the lysosomal membrane and releases proteases into the cytoplasm, triggering apoptosis and necrosis (Zhang et al., 2021). In liver, Kupffer cells are a major source of ROS, which is normally produced by nicotinamide adenine dinucleotide phosphate (NADPH) oxidase. ATP activates Kupffer cells leading to ROS generation. Some lipid peroxidation products could activate hepatic stellate cells; in turn, hepatic stellate cells could produce ROS. During ER stress, mitochondrial ROS production increases, and Ca^2+^ leaked from the ER is taken up by mitochondria. Ca^2+^ uptake leads to the release of cytochrome C, which further eliminates the electron transport chain function and leads to increased ROS overproduction (Jangra et al., 2022).

Lipotoxicity is one of the major mechanisms of hepatocyte dysfunction and contributes to the progression of NASH (Horn et al., 2022). Previous studies have suggested that triglyceride accumulation in hepatocytes could cause NALFD, followed by oxidative stress and lipid peroxidation, promoting inflammation and fibrosis (Svegliati-Baroni et al., 2019). However, this argument has been challenged in animal models and clinical trials in recent years. There were data suggesting that triglyceride accumulation may actually be a protective mechanism against lipotoxicity. For example, inhibition of triglyceride synthesis in mice reduced hepatic steatosis but exacerbated liver injury and fibrosis (Newberry et al., 2017). Furthermore, triglyceride accumulation was not sufficient to cause inflammation in the liver, whereas other lipotoxic metabolites were a major contributor to disease progression. In general, animal fats are rich in saturated fatty acids palmitate and stearate, which are directly toxic to cells (Packard et al., 2020), while monounsaturated fatty acids could prevent saturated fatty acid-induced cytotoxicity (Urso and Zhou, 2021). Saturated fatty acids could induce hepatic endoplasmic reticulum stress and promote inflammasome activation, which could also activate amino-terminal kinase (JNK) and mitochondrial death pathways to induce hepatocyte apoptosis (Luci et al., 2020; Zhu et al., 2022). Increased hepatocyte free fatty acids could form lipotoxic intermediates in the liver, such as ceramides, diacylglycerols, lysophosphatidylcholines, oxidized fatty acids, and cholesterol metabolites, which act like ROS (Sowton et al., 2020). In addition to saturated fatty acids, excess cholesterol could also induce hepatotoxicity and be associated with NASH progression (Coué et al., 2019). The cellular damaging effects of cholesterol were primarily mediated by sensitizing cells to other cell death signals. Excessive free cholesterol accumulated in hepatocytes, Kupffer cells, hepatic stellate cells, disrupts membrane fluidity and caused glutathione loss in mitochondria, leading to oxidative stress, mitochondrial dysfunction and ATP depletion, and cellular apoptosis and necrosis. In addition, cholesterol crystals have been reported to be present in the lipid droplet membranes of hepatocytes *in vivo* and clinical NASH (Farrell et al., 2018; Horn et al., 2022). These crystals activated the inflammasome and acted in Kupffer cells and macrophages, leading to the production of IL-1β and TNF (Ioannou et al., 2017; Wree et al., 2018). Taken together, Kupffer cells and hepatic stellate cells could accumulate free cholesterol, thereby activating signaling of liver inflammation and fibrosis, leading to the development of NAFLD to NASH.

### 3.2 Mitochondrial dysfunction

In the process of NAFLD progression to NASH, mitochondrial dysfunction is one of the important mechanisms. One of the main causes of mitochondrial deterioration in NASH is fatty acid oxidation and lipotoxicity (Carnagarin et al., 2021). The ever-increasing flux of fatty acids through mitochondria and the TCA cycle generated harmful ROS that attack the mitochondrial respiratory chain and mitochondrial DNA, and inhibit mitochondrial respiratory chain complex activity (Mooli and Ramakrishnan, 2022). Over time, mitochondria gradually became dysfunctional, triggering oxidative stress, ATP depletion, and disruption of mitochondrial integrity, all of which lead to hepatocyte death (Manne et al., 2018). Interestingly, both mitochondrial DNA and oxidized mitochondrial DNA released into the cytoplasm during programmed cell death cause inflammasome activation. These results provided a link between mitochondrial dysfunction, apoptosis, and inflammasome activation.

### 3.3 Nuclear receptors

Free fatty acids derived from adipose tissue breakdown serve as natural nuclear receptor ligands and play a major role in the regulation of NASH-related lipid metabolism and inflammation (Yadati et al., 2021). Polyunsaturated fatty acids and their derivatives could activate peroxisome proliferator-activated receptor-α (PPARα), which could induce the expressions of genes involved in fatty acid oxidation and insulin sensitivity, and downregulate nuclear factor κB (NF-κB) level (Geethangili et al., 2021; López-Salazar et al., 2021). However, in a mouse model of NASH, a diet fed polyunsaturated fatty acids activates PPARα signaling and inhibits hepatic *de novo* fat synthesis, but does not prevent steatohepatitis (Ishida et al., 2021), which is likely to high levels of hepatic lipid peroxidation in this model abolished the protective effect of PPARα activation and resulted in lipotoxic hepatocyte injury and inflammatory cells recruitment. Similar to PPARα, PPARγ activation attenuates inflammation through multiple mechanisms, including decreased NF-κB activity and decreased TNF-α and IL-1β synthesis in monocytes and macrophages (Liu et al., 2022). As a master regulator of macrophage polarization, activation of PPARγ promotes the transition of macrophages from an M1-dominant phenotype to an M2 phenotype, thereby attenuating inflammation in experimental NASH (Luo et al., 2017; Valenzuela et al., 2017). Furthermore, PPARδ directly controls the inflammatory response of Kupffer cells in the liver. Other members of the nuclear receptor family, such as liver X receptors (LXRs), are also critical for the control of fatty acid synthesis and inflammation (Cai et al., 2017; Welch et al., 2022).

## 4 Outlook

Taken together, two factors, intrahepatic and extrahepatic, could trigger the transition from isolated steatosis to NASH, and especially, determining the important role of hepatic inflammatory response during this process. Lipotoxicity causes cell apoptosis through peroxidative stress, endoplasmic reticulum stress, autophagy, etc., resulting in liver cell damage or death, and further leads to insulin resistance, which is the dominant factor in fat metabolism disorders. A vicious cycle of hepatic fat accumulation-liver injury-insulin resistance-lipid metabolism disorder. In addition, the imbalance of intestinal microbial homeostasis leads to the production of a large number of bacterial products, including short-chain fatty acids, lipopolysaccharides, deoxyribonucleic acid, polyamine metabolites, resulting in the accumulation of a large amount of fat in the liver, causing liver cell injury, inflammatory response, insulin resistance and liver fibrosis. On the contrary, the inflammatory response triggered by liver cell injury could chemotactic the infiltration of monocytes and lymphocytes, and participate in the formation and development of liver inflammation. Recently, although substantial progress has been made in revealing the mechanisms of NASH development, there are still no effective remedies available for alleviating or treating the disease. This requires further studies with integrated metabolomic, proteomic and epigenetic biology approaches to reveal the molecular features of NASH, searches for biomarkers of NASH, and identify more rational strategies.
